# Determinants of male reproductive health disorders: the Men in Australia Telephone Survey (MATeS)

**DOI:** 10.1186/1471-2458-10-96

**Published:** 2010-02-24

**Authors:** Carol A Holden, Robert I McLachlan, Marian Pitts, Robert Cumming, Gary Wittert, Johnathon P Ehsani, David M de Kretser, David J Handelsman

**Affiliations:** 1 Andrology Australia, Monash Institute of Medical Research, Monash University, Melbourne, Victoria, Australia; 2 Prince Henry's Institute, Melbourne, Victoria, Australia; 3 Australian Research Centre in Sex, Health and Society, La Trobe University, Victoria, Australia; 4 Centre for Education & Research on Ageing and School of Public Health, University of Sydney, Sydney, NSW, Australia; 5 Department of Medicine, University of Adelaide, Adelaide, SA, Australia; 6 Formerly: Department of Human Services, Victoria, Australia; 7 Monash Institute of Medical Research, Monash University, Melbourne, Victoria, Australia; 8 Department of Andrology, Concord Hospital, ANZAC Research Institute, University of Sydney, Sydney, NSW, Australia

## Abstract

**Background:**

The relationship between reproductive health disorders and lifestyle factors in middle-aged and older men is not clear. The aim of this study is to describe lifestyle and biomedical associations as possible causes of erectile dysfunction (ED), prostate disease (PD), lower urinary tract symptoms (LUTS) and perceived symptoms of androgen deficiency (pAD) in a representative population of middle-aged and older men, using the Men in Australia Telephone Survey (MATeS).

**Methods:**

A representative sample (n = 5990) of men aged 40+ years, stratified by age and State, was contacted by random selection of households, with an individual response rate of 78%. All men participated in a 20-minute computer-assisted telephone interview exploring general and reproductive health. Associations between male reproductive health disorders and lifestyle and biomedical factors were analysed using multivariate logistic regression (odds ratio [95% confidence interval]). Variables studied included age, body mass index, waist circumference, smoking, alcohol consumption, physical activity, co-morbid disease and medication use for hypertension, high cholesterol and symptoms of depression.

**Results:**

Controlling for age and a range of lifestyle and co-morbid exposures, sedentary lifestyle and being underweight was associated with an increased likelihood of ED (1.4 [1.1-1.8]; 2.9 [1.5-5.8], respectively) and pAD (1.3 [1.1-1.7]; 2.7 [1.4-5.0], respectively. Diabetes and cardiovascular disease were both associated with ED, with hypertension strongly associated with LUTS and pAD. Current smoking (inverse association) and depressive symptomatology were the only variables independently associated with PD. All reproductive disorders showed consistent associations with depression (measured either by depressive symptomatology or medication use) in both age-adjusted and multivariate analyses.

**Conclusion:**

A range of lifestyle factors, more often associated with chronic disease, were significantly associated with male reproductive health disorders. Education strategies directed to improving general health may also confer benefits to male reproductive health.

## Background

Several studies have investigated a range of overlapping risk factors in relation to male reproductive health. These include socio-demographic characteristics (such as age, education, socio-economic status), lifestyle factors (such as alcohol consumption, smoking, physical activity) and biomedical risk factors (such as heart disease, diabetes, drug therapies) [1-3].

Age is consistently associated with increased prevalence of lower urinary tract symptoms (LUTS), erectile dysfunction (ED) and benign prostatic hyperplasia (BPH) [1,4,5]. While some older men may consider poorer reproductive health a consequence of the ageing process [4-6], younger men express high levels of concern about developing reproductive health problems in later years [4].

Most studies focus on ED as the predominant male reproductive health disorder, with evidence suggesting that lifestyle risk factors (such as obesity and sedentary lifestyles) for ED are similar to those for cardiovascular disease and diabetes [7,8]. These suggestions are based on observations that men with other such co-morbid conditions also report significantly higher rates of ED [8]. It has therefore been proposed that the assessment of erectile function may provide a useful indicator for earlier detection and treatment of other life-threatening conditions [9]. Indeed, the association between obesity and ED has recently been explored with sexual function being improved in a proportion of obese men after lifestyle changes to reduce total body weight [10]. However, the relationship between other reproductive health disorders and a range of behavioural and biomedical determinants is less clear.

Limitations exist in the comparison of studies that focus on individual reproductive health disorders in isolation due to methodological differences, particularly due to sampling from different populations and different definitions used in the reporting of prevalence rates. This national, population-based cross-sectional study explored a range of behavioural and biomedical factors and their association with reproductive health disorders in a representative sample of Australian men aged 40 years and over. By investigating a range of reproductive health disorders within the same population of men, commonality between health conditions and health determinants can be more clearly identified.

## Methods

The MATeS study has been described previously [4]. In summary, between September and December 2003, computer-assisted telephone interviews (CATIs) of a representative sample of men from across Australia, aged 40 years and over, were undertaken. Within each State and Territory, respondents were stratified into four age groups: 40-49 years, 50-59 years, 60-69 years and 70+ years. Sampling continued until a minimum of 1250 men in each age stratum had been surveyed.

Respondents were recruited from across all States and Territories in Australia by random selection of households from the electronic telephone directory (Electronic White Pages, EWP), with over sampling in some age brackets and geographical regions to ensure proportionate representation. Information was sent to households describing what was required of participants to be in the study and the inclusion criteria. The interviewer phoned the households and asked potential participants if they were willing to be interviewed. An affirmative response to participation was taken as consent. An overall individual response rate of 78% was achieved. Socio-demographic characteristics of the men participating in the study were generally representative of the Australian population [4]. The study was approved by the Southern Health Human Research Ethics Committee, Melbourne.

### Telephone survey instrument

The telephone survey instrument included more than 90 questions focusing on socio-demographic issues (age, marital status, ethnicity, occupation and education), general health and lifestyle, sexual function, fertility, contraception, relationship issues, and knowledge, attitudes and beliefs concerning male reproductive health and its disorders. The survey took 17.5 minutes, on average, to complete. Men in the youngest age group completed interviews in a shorter average time than those in the oldest age group (16.4 and 18.5 minutes, respectively). The full CATI is available at http://www.andrologyaustralia.org/docs/Methods_Manual_CATI.pdf

### Outcomes

All information obtained from the interview was self-report with no check against medical records. Where available, questions from other validated instruments and those used in an Australian context were incorporated. Using the Massachusetts Male Aging Study (MMAS) single question [11], erectile dysfunction was defined as 'sometimes' (moderate) or 'never' (severe) being able to get and keep an erection that is rigid enough for satisfactory sexual activity. LUTS were assessed using the International Prostate Symptom Score (IPSS) and defined as absent or mild (score ≤ 7), moderate (score 8-19), or severe (score 20-35) [12]. A self-reported diagnosis of prostate disease (PD) was indicated if the respondent noted that a doctor had ever told them they had a prostate problem. The question 'Do you think you may currently be suffering from low levels of testosterone now?' was included to assess men's perception of whether they suffer from symptoms of androgen deficiency. A positive response was coded as 'perceived symptoms' of androgen deficiency (pAD). Self-reports of specific diseases were noted when men reported receiving a specific diagnosis or prescribed medication by a doctor.

### Exposures

Exposure variables were all self-reported including height, weight and waist circumference. Self-reported waist circumference has been shown to have reasonable validity and self-reported weight to be highly correlated with measured weight [13].

#### Lifestyle factors

Body Mass Index (BMI) was classified as underweight (<20 kg/m^2^); acceptable (20.0-24.9 kg/m^2^); overweight (25.0-29.9 kg/m^2^); and obese (≥ 30 kg/m^2^). Waist circumference was classified as small (<94.0 cm); medium (94.0-101.9 cm); and large (≥ 102 cm) [14]. Cigarette (or other tobacco product) smoking, alcohol consumption and physical activity (assessed by recall of intensity, frequency and type in the last week) were classified according to Australian guidelines [15-17]. The categories are described in Table 1.

**Table 1:** Age-adjusted associations of lifestyle factors with male reproductive health problems: OR (95% CI)

Characteristic	Number of men in each category^a^	ED	LUTS	PD	pAD
**Prevalence of condition**		21.3% (1012/4753^a^)	16% (811/4986)	14% (677/4993)	31% (1172/3756)

**Waist circumference (cm)**					
Small (<94)	2470	0.7 (0.6-0.9)*	0.9 (0.7-1.1)	1.0 (0.8-1.2)	0.7 (0.6-0.9)**
Medium (94.0-101.9)	939	1.0 (referent)	1.0 (referent)	1.0 (referent)	1.0 (referent)
Large (≥ 102)	952	1.3 (1.0-1.6)	1.1 (0.9-1.3)	1.1 (0.9-1.4)	1.2 (1.0-1.5)
**BMI (kg/m^2^)**					
Underweight (<20)	111	2.4 (1.3-4.3)*	0.8 (0.5-1.3)	0.6 (0.4-1.0)	2.1 (1.2-3.8)
Acceptable (20.0-24.9)	1652	1.0 (referent)	1.0 (referent)	1.0 (referent)	1.0 (referent)
Overweight (25.0-29.9)	2282	1.1 (0.9-1.3)	0.9 (0.8-1.1)	0.9 (0.7-1.0)	1.2 (1.0-1.4)
Obese (≥ 30)	885	1.8 (1.4-2.2)**	1.2 (0.9-1.4)	0.8 (0.6-1.0)	1.8 (1.5-2.3)**
**Alcohol consumption**					
Abstainer^†^	630	1.0 (referent)	1.0 (referent)	1.0 (referent)	1.0 (referent)
Low risk drinker^‡^	4002	0.7 (0.6-0.9)*	0.7 (0.6-0.9)*	1.0 (0.8-1.3)	0.9 (0.7-1.1)
Risky or high risk drinker^§^	359	1.1 (0.8-1.6)	0.9 (0.6-1.3)	0.7 (0.4-1.0)	1.4 (1.0-1.9)
**Cigarette smoking**					
Current smoker^||^	912	1.3 (1.0-1.6)	1.1 (0.9-1.4)	0.6 (0.4-0.8)*	1.3 (1.1-1.7)*
Former smoker^¶^	2321	1.2 (1.0-1.4)	1.0 (0.9-1.2)	1.0 (0.8-1.2)	1.3 (1.1-1.5)*
Never smoked	1769	1.0 (referent)	1.0 (referent)	1.0 (referent)	1.0 (referent)
**Physical Activity**					
Sedentary^§§^	1166	1.5 (1.2-1.8)**	1.1 (0.9-1.4)	0.8 (0.6-1.0)	1.3 (1.1-1.6)*
Insufficient activity^††^	2075	1.1 (0.9-1.3)	1.0 (0.8-1.1)	1.0 (0.8-1.2)	1.1 (0.9-1.3)
Sufficient activity^‡‡^	1749	1.0 (referent)	1.0 (referent)	1.0 (referent)	1.0 (referent)

^a ^Note: numbers included in each analysis differ slightly from those presented here due to missing data.

* p < 0.01 ** p < 0.001

^† ^Do not consume alcohol; ^‡ ^Drink up to 6 standard drinks on any one day, no more than three days a week; ^§ ^Risky drinkers (7 to 10 standard drinks on any one day) and high risk drinkers (11 or more standard drinks on any one day)

^||^Currently smoke, daily, weekly or less often than weekly; ^¶ ^Do not smoke now but have smoked at least 100 cigarettes or equivalent tobacco over a lifetime

^§§ ^No participation in physical activity; ^†† ^Some physical activity reported but not meeting 'sufficient' criteria; ^‡‡ ^At least five separate sessions of vigorous-intensity activity

#### Co-morbid conditions

Men were asked if they had ever been told by a doctor that they had the following illnesses: high blood pressure ever requiring treatment (hypertensive disease), angina or heart attack (cardiovascular disease), stroke (cerebrovascular disease), or diabetes ever requiring treatment. Only men who reported a diagnosed prostate problem were asked if they had undertaken surgery to treat the prostate problem. Depressive symptomatology was reported when men gave a positive response to the question 'In the last 12 months, have you been depressed enough to interfere with daily living?'

#### Medications

All men were asked if they are currently taking tablets for high blood pressure and/or high cholesterol and whether they had ever taken medication for depression.

### Data analysis

Descriptive analyses have been reported previously [4]. All analyses were done using data weighted according to age and state of residence, based on the 2001 census age distribution of Australian men [18]. Binary logistic regression was used to explore associations between each health outcome (ED, LUTS, PD and pAD) and each lifestyle factor, co-morbid condition and medication used, controlling for age in the first instance. For ED analyses, the moderate and severe categories were combined. Moderate and severe categories of LUTS were also combined (IPSS scores 8-35) for logistic regression. Lifestyle factors included waist circumference groups, BMI categories, alcohol consumption, cigarette smoking and physical activity. Co-morbid conditions included cerebrovascular disease, cardiovascular disease, hypertensive disease, diabetes requiring treatment, depressive symptomatology and prostate surgery. Medications included use of medications for depression, high blood pressure and/or high cholesterol.

Further logistic regression analyses were performed to assess independent associations of lifestyle factors, co-morbid conditions and medication use with each health outcome, controlling for age as well as all lifestyle variables, co-morbid conditions and medication use (fully adjusted models). Odds ratios (ORs) and 95% confidence intervals (95% CIs) are presented in the results. Due to multiple comparisons p < 0.01 was used as the criterion for statistical significance. Logistic regression analyses were performed using Stata 8.0 (Stata Corporation, College Station, Texas).

## Results

### Association of lifestyle factors with male reproductive health disorders

#### Waist Circumference

**Figure 1:**
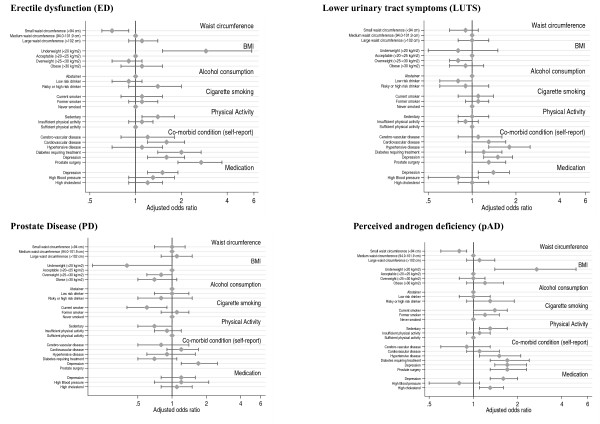
**Associations of male reproductive health disorders with a range of lifestyle factors and biomedical determinants**. Associations of male reproductive health disorders with a range of lifestyle factors and biomedical determinants in middle-aged and older Australian men. Each model controls for age, waist circumference, BMI, smoking, alcohol consumption, physical activity, co-morbid disease and medication use for high blood pressure, high cholesterol and depression. Adjusted ORs and 95% CIs are presented.

When controlling for age only, men with a waist circumference of < 94 cm were significantly less likely to report erectile problems (ED) and perceived androgen deficiency (pAD) while those with a large waist circumference (>102 cm) had a higher likelihood of ED compared to men in the medium waist circumference category but this did not reach statistical significance (Table 1). When each outcome variable was adjusted for all lifestyle factors, co-morbid diseases and medication use (Figure 1), the strength of the association between large waist circumference and ED was reduced. Waist circumference was not associated with either LUTS or prostate disease.

#### Body Mass Index (BMI)

Underweight men were more likely to report erectile problems (ED) and pAD in age-adjusted and fully adjusted models. Obesity was positively associated with ED and pAD in age-adjusted analyses (Table 1) but in fully adjusted models obesity was not significantly associated with any reproductive health disorders (Figure 1).

#### Alcohol consumption

Moderate alcohol consumption was associated with lower risk of ED and LUTS when controlling for age (Table 1) but these associations were attenuated in the fully adjusted models and the confidence intervals included the null value (Figure 1).

Excessive alcohol consumption as defined by high risk drinking behaviour was not significantly associated with any reproductive health disorder compared to the abstaining group (Table 1 & Figure 1).

#### Smoking

Adjusting for age, smoking (either current or former) showed no significant association with ED or LUTS (Table 1). However, both current and former smokers had a modestly increased likelihood of reporting pAD, although in the fully adjusted model the confidence intervals included the null value. Current smokers were less likely than 'never smokers' to report PD in both the age-adjusted and fully adjusted models (Table 1 & Figure 1).

#### Physical Activity

Compared to men with sufficient levels of physical activity, sedentary men had a higher risk of ED and pAD. An upper confidence limit of 1.0 for the reduced likelihood of sedentary men reporting PD suggests the evidence for this association is weak (Table 1 & Figure 1). The 'insufficient physical activity' category did not show associations with male reproductive health problems (Figure 1).

### Association of co-morbid conditions and medication use with male reproductive health disorders

After controlling for age, men with self-reported cerebrovascular disease, cardiovascular disease, hypertensive disease, diabetes and depressive symptomatology (in the last 12 months) were more likely to report ED, LUTS and pAD than men without these conditions, although the CI around the OR of 1.3 for pAD in men with cerebrovascular disease included the null value (Table 2). In general, the associations with these co-morbid conditions were stronger for ED, with several ORs greater than 2, than for the other male reproductive disorders. After controlling for all other factors diabetes remained associated with ED and pAD and associations between cardiovascular disease and ED persisted (Figure 1).

**Table 2:** Age-adjusted associations of biomedical factors with male reproductive health problems: OR (95% CI)

Characteristic	Number of men with the condition/taking medication^a^	ED	LUTS	PD	pAD
**Co-morbid condition (self-report)**					
Cerebrovascular disease	177	2.1 (1.5-3.0)*	1.5 (1.1-2.1)*	1.0 (0.7-1.4)	1.3 (0.9-1.9)
Cardiovascular disease	559	2.0 (1.6-2.5)**	1.4 (1.2-1.7)**	1.2 (1.0-1.4)	1.5 (1.2-1.8)**
Hypertensive disease	1294	1.6 (1.3-1.8)**	1.5 (1.3-1.7)**	1.1 (1.0-1.4)	1.5 (1.3-1.8)**
Diabetes requiring treatment	345	2.8 (2.1-3.7)**	1.4 (1.1-1.8)*	0.8 (0.7-1.1)	2.2 (1.7-2.8)**
Depressive symptomatology	640	1.8 (1.4-2.3)**	1.7 (1.4-2.1)**	1.6 (1.2-2.1)**	2.1 (1.7-2.7)**
Prostate surgery	303	3.0 (2.3-4.0)**	1.3 (1.0-1.7)	--	1.8 (1.4-2.3)**
**Medication**					
Depression	706	1.9 (1.5-2.3)**	1.7 (1.4-2.1)**	1.5 (1.2-1.8)**	2.1 (1.7-2.5)**
High Blood pressure	1218	1.7 (1.5-2.0)**	1.3 (1.1-1.5)*	1.2 (1.0-1.5)	1.4 (1.2-1.6)**
High cholesterol	857	1.8 (1.5-2.1)**	1.3 (1.1-1.5)*	1.2 (1.0-1.5)	1.6 (1.4-1.9)**

^a ^Note: numbers included in each analysis differ slightly from those presented here due to missing data.

* p < 0.01 ** p < 0.001

The age-adjusted associations between self-reported depressive symptomatology and all reproductive health disorders (Table 2) persisted in the fully adjusted models (Figure 1). The associations between depression medication and reproductive health conditions were similar to those for self-reported depressive symptomatology.

While self-reported hypertensive disease was associated with LUTS, and pAD in fully adjusted models, medication use for high blood pressure was not associated with any reproductive health disorder (Figure 1). With the exception of depressive symptomatology, the reporting of co-morbid conditions and use of the medications we studied were not significantly associated with PD (Figure 1).

Prostate surgery was associated with ED and pAD, in both age-adjusted (Table 2) and fully adjusted models (Figure 1).

## Discussion

With a wide-ranging approach that considers male reproductive health as a whole, this large, cross-sectional and population-based study provides a more complete picture than previous studies of the associations between lifestyle and clinical factors and male reproductive health disorders, notably ED, LUTS, prostate disease (PD) and symptoms of perceived androgen deficiency (pAD). As the first ever national stratified random sample survey of older men, it has provided information on male reproductive health and associated factors that is more robust and generalisable than that from previous smaller clinical cohorts or even large population based studies confined to single cities or regions. The study highlights relationships between a range of reproductive health disorders and general health issues in men that would not be evident when viewing reproductive health problems in isolation.

Associations between ED and lifestyle risk factors similar to those for cardiovascular disease (such as waist circumference and physical activity) were confirmed in our study. Such associations also extended to other reproductive health disorders, with multivariate logistic regression analysis revealing that a sedentary lifestyle was also an independent risk factor for pAD. The relationship between physical inactivity and lower risk of PD was not explained by adjustment for other lifestyle factors and co-morbid conditions, such as via a relationship between obesity and lowered blood testosterone concentrations, and remains enigmatic [19]. In contrast to previous studies [7,20,21], the effects of obesity on reproductive health were diminished when adjusted for other lifestyle and co-morbid conditions, suggesting that other factors are confounding this effect. Sedentary lifestyle may be the predetermining factor, irrespective of obesity whereas previous studies have suggested that increased physical activity is associated with less adverse male reproductive health, including erectile dysfunction [22], prostate disease and lower urinary tract symptoms [19]. In contrast to our findings, obesity has been linked with more frequent benign [23] and malignant prostate disease [24,25].

Another interesting observation is the higher likelihood of adverse reproductive health status with being underweight (BMI < 20 kg/m^2^), although in our study this represents a small group of men. This may be attributable to lower weight as a consequence of serious underlying disease and/or possibly a reduced blood testosterone as suggested by an unanticipated protective role for prostate disease. Cheng et al [26] also identified a U-shaped relationship between BMI and ED with underweight men having a higher risk than men of normal weight but this was only evident in men who did not exercise. The novel associations of underweight and sedentary lifestyle with increased risks of ED and of pAD and a reduced risk of prostate disease warrant further analysis.

Smoking was associated with reduced risk of PD which is consistent with several reports that smokers have fewer operations for benign prostatic hyperplasia (BPH) or report fewer symptoms of BPH [1,27]. Whether this is related to a genuine underlying biological mechanism such as that mediated by alteration in sex hormone metabolism in smokers [28], although others have found no association [29], or whether it reflects indirectly the operation of other unrecognised health factors influencing both smoking status and PD, remains to be clarified. In contrast, evidence to suggest that smoking [20,30] and alcohol consumption [8] increase the risk of ED were not confirmed in this study. Alcohol consumption appears to have no independent effect on the range of reproductive health disorders examined in this study.

The strong association between ED and cardiovascular disease and diabetes supports several previous studies suggesting that common mechanisms exist between reproductive problems and concurrent disease [3,31-33]. Associations with cerebrovascular disease and hypertensive disease identified in age-adjusted analyses have been shown in other studies [3,20], although in our study these associations were reduced in multivariate analysis. Similarly, strong associations between LUTS and cardiovascular disease and treatment of hypertension have been demonstrated [34]. These associations again highlight that reproductive health problems co-exist, or may be considered as clinical markers, with other co-morbid disease. This study supports suggestions that assessment of erectile function in middle-aged and older men may provide a useful indicator to modify lifestyle factors and reduce the risk of other life-threatening conditions [10,35,36].

One striking finding in this study is the similarity of odds ratios for ED and pAD across virtually all variables. The risk factor similarities are difficult to explain in biological terms as low blood testosterone levels are an uncommon primary cause of erectile failure but the lack of libido may be confused with ED [37]. Participants may report suffering from perceived androgen deficiency when confused with non-specific feelings of poorer health and/or confusion of declining sexual function with symptoms of androgen deficiency [4]. There are acknowledged difficulties with measuring androgen deficiency in a study of this type and instruments are known to have low specificity [38]. If an interpretation of poorer health and/or declining sexual function is applied, then the risk factor associations identified can be more clearly explained. It is well known that chronic diseases in general, including most common co-morbid conditions associated with ageing, have a non-specific effect to lower blood testosterone concentrations [39,40]. The observation also suggests that middle-aged Australian men in this study may identify androgen deficiency as synonymous with erectile failure. At the time the study was done there had been little legitimate effort to raise the awareness of the public (and medical profession) about low testosterone and the symptoms or circumstances that should raise concern. The observed semantic and biological confusion indicates that community education is important both to clarify men's understanding of their healthy ageing as well as to stem the tide of androgen over-prescribing which is escalating in some countries [41].

An important novel observation from this study is the strong association between depressive symptomatology and all reproductive health disorders assessed, which persisted when all other risk factors (age, lifestyle and biomedical determinants) were taken into consideration. While studies have demonstrated associations between ED and depression previously [42,43], this is the first study to demonstrate the strong association with all reproductive health disorders examined in the one sample population. This observation highlights the effect of reproductive health problems on quality of life, as potentially manifest by psychosocial factors.

The findings from this study are not unique to Australian men as evidenced by studies from a range of other populations and clinical cohorts in different settings, where various aspects of our findings have been reported [7,44-47]. As for any observational study, the associations identified cannot confer causality or affirm its direction (if any), and the role of unmeasured confounding may be influencing these effects. Nevertheless the consistency of association between lifestyle and biomedical factors over a wide range of male reproductive health disorders suggests a definite underlying pattern for which only more complex longitudinal and interventional studies can fully define. This study therefore highlights the importance of longitudinal and/or interventional studies to determine the causality of potential risk factors and identify possible preventative measures to retard or reverse disease progression.

Similarly, the present observational study suggests that maintaining general health with beneficial lifestyle behaviours that are more often associated with other co-morbid disease, such as physical activity, may confer benefits for reproductive health of middle-aged and older men, a concept that warrants further direct study. Physical activity appears to confer benefits in sexual function among older men, with physically active older people reporting higher rates of sexual activity and sexual satisfaction than their less active counterparts [48]. Similarly, studies suggest that physically active older adults report lower rates of depressive illness [49,50]. If the direction of causality in the associations identified in this study were verified, such as via interventional or longitudinal studies, the interaction of physical activity, reproductive health and depression could be further investigated as such associations may provide the basis of education campaigns that benefit many aspects of health and well-being in the middle-aged and older population.

## Conclusions

The study highlights that reproductive health should not be considered in isolation from general health in middle-aged and older men. If opportunities to identify reproductive health disorders are being missed [4-6], the risk of more life-threatening conditions may also be left undetected. Identification of modifiable risk factors that contribute to chronic disease but also to a possible decline in reproductive health with age will be essential for the development of appropriate preventative strategies and will better inform health policy development and service provision for the progressively ageing population. Education for both the community and the professions is imperative to ensure that middle-aged and older men know when to seek treatment for reproductive health problems and receive appropriate management when accessing health services. Finally, the strong association with cardiovascular disease and depression (depressive symptomatology and medication use) for a range of reproductive health disorders highlights the importance of including male reproductive health as both a sentinel marker and an outcome in other chronic disease studies.

## Competing interests

The authors declare that they have no competing interests.

## Authors' contributions

All authors contributed to study design; survey development, checking and analysing data; and took part in interpretation of findings and drafting and preparation of the manuscript. CAH had full access to all of the data in the study and takes responsibility for the integrity of the data and the accuracy of the data analysis. JPE performed logistic regression analysis. All authors have read and approved the final manuscript.

## Pre-publication history

The pre-publication history for this paper can be accessed here:

http://www.biomedcentral.com/1471-2458/10/96/prepub
